# Immunoprofiling Correlates of Protection Against SHIV Infection in Adjuvanted HIV-1 Pox-Protein Vaccinated Rhesus Macaques

**DOI:** 10.3389/fimmu.2021.625030

**Published:** 2021-05-11

**Authors:** Pinyi Lu, Dylan J. Guerin, Shu Lin, Sidhartha Chaudhury, Margaret E. Ackerman, Diane L. Bolton, Anders Wallqvist

**Affiliations:** ^1^ Biotechnology HPC Software Applications Institute, Telemedicine and Advanced Technology Research Center, U.S. Army Medical Research and Development Command, Fort Detrick, MD, United States; ^2^ Henry M. Jackson Foundation for the Advancement of Military Medicine, Rockville, MD, United States; ^3^ Thayer School of Engineering, Dartmouth College, Hanover, NH, United States; ^4^ Center for Enabling Capabilities, Walter Reed Army Institute of Research, Silver Spring, MD, United States; ^5^ U.S. Military HIV Research Program, Walter Reed Army Institute of Research, Silver Spring, MD, United States

**Keywords:** adjuvanted HIV-1 vaccine, systems serology, Fc receptor, immune correlate, rhesus macaque

## Abstract

Human immunodeficiency virus type 1 (HIV-1) infection remains a major public health threat due to its incurable nature and the lack of a highly efficacious vaccine. The RV144 vaccine trial is the only clinical study to date that demonstrated significant but modest decrease in HIV infection risk. To improve HIV-1 vaccine immunogenicity and efficacy, we recently evaluated pox-protein vaccination using a next generation liposome-based adjuvant, Army Liposomal Formulation adsorbed to aluminum (ALFA), in rhesus monkeys and observed 90% efficacy against limiting dose mucosal SHIV challenge in male animals. Here, we analyzed binding antibody responses, as assessed by Fc array profiling using a broad range of HIV-1 envelope antigens and Fc features, to explore the mechanisms of ALFA-mediated protection by employing machine learning and Cox proportional hazards regression analyses. We found that Fcγ receptor 2a-related binding antibody responses were augmented by ALFA relative to aluminium hydroxide, and these responses were associated with reduced risk of infection in male animals. Our results highlight the application of systems serology to provide mechanistic insights to vaccine-elicited protection and support evidence that antibody effector responses protect against HIV-1 infection.

## Introduction

The HIV-1 AIDS epidemic remains a major public health threat, claiming over half a million lives globally annually (1). An efficacious HIV-1 vaccine is considered the most effective tool to halt the ongoing HIV-1 epidemic (2). To date, the Thai phase 3 HIV vaccine trial RV 144 was the only trial to demonstrate efficacy against HIV acquisition, with 60.5% and 31.2% efficacy one and three years following vaccination, respectively (3, 4). The follow up HVTN 702 trial evaluating a similar pox-protein HIV vaccine regimen did not recapitulate the efficacy observed in RV 144. However, as numerous parameters differed between these two clinical studies, and several pre-clinical animal studies have supported hypotheses generated by the RV 144 findings, the results of RV 144 remain valid and warrant continued investigation. Therefore, continuous and significant efforts are still required for developing a safe and more effective HIV-1 vaccine.

To improve and sustain HIV vaccine efficacy, multiple novel strategies are being pursued. These include evaluation of other viral vectors, such as adenovirus serotype 26 and cytomegalovirus, and adjuvants. Aluminum salts (alum) are the classical adjuvant and are employed in most licensed vaccines (5). Novel vaccine adjuvants are an active area of product development and have been adopted for vaccines against multiple pathogens. Liposomal adjuvants are particularly promising, as exemplified by the highly successful Shingrix zoster vaccine. We recently evaluated a liposomal adjuvant, ALFA, for HIV-1 Env protein vaccination in combination with pox vector priming for efficacy against SHIV acquisition in rhesus macaques (6). ALFA, or Army Liposome Formulation adsorbed to aluminum hydroxide, consists of liposomes containing saturated phospholipids, cholesterol, and monophosphoryl lipid A, and has exhibited excellent safety and potency in clinical trials (7). Adjuvanting with ALFA reduced the per-exposure SHIV infection risk by 59% compared to controls, while adjuvanting with aluminum hydroxide did not protect against infection. Significant sex differences were observed, with vaccine efficacy limited to male animals (90%). Antibody-dependent neutrophil and monocyte phagocytotic responses, but not binding antibody responses, were increased by ALFA relative to alum, and these responses correlated with protection. Neutralizing antibody responses were robust and comparable between the two active arms, but limited to tier 1. The underlying mechanism(s) for ALFA-mediated protection against infection and augmented phagocytotic responses are unclear.

In the present study, we evaluated a broad range of antibody characteristics relevant to non-neutralizing antibody functions as assessed by an Fc array assay measuring Fv and Fc characteristics of antibodies in the vaccinated macaques. We aimed to determine the immune signature of different adjuvant formulations in a nonhuman primate HIV vaccine model and reveal the underlying mechanism linked to the observed ALFA-enhanced phagocytotic responses. In line with previous findings from hierarchical clustering and principal component analysis (6), our results showed a large overlap in the immune signatures of ALFA- and alum-adjuvanted vaccines, consistent with the overall similar vaccine regimens. The main aspects of variation in the data did not relate to adjuvants, yet differential protection was observed between adjuvants. Thus, we next sought to identify differences in individual immune features that were associated with adjuvants using univariate analysis. We found that ten Fc receptor-related immune responses were significantly enhanced by the vaccine adjuvanted with ALFA compared to alum. We then trained random forest models to determine which adjuvant-associated immune responses can best discriminate two adjuvant formulations on an individual level. Finally, we used a Cox regression analysis to determine whether immune responses most predictive of adjuvant, as identified by random forest models, were associated with reduced risk of infection over time. Among the ten ALFA-specific immune responses, three Fcγ receptor 2a-mediated immune responses strongly correlated with protection, but only in males. Our approach integrating univariate analysis, machine learning, and Cox regression analysis was effective in analyzing high-dimensional immune data and capable of identifying immune features associated with vaccine efficacy and inferring vaccine protection mechanisms.

## Methods

### Immunization and SHIV Challenge of Rhesus Macaques

An HIV-1 vaccine NHP study was performed as previously described (6). Briefly, 48 rhesus macaques were assigned to three arms that were balanced across multiple factors, including *TRIM*5 alleles*, TRIMcyp* positivity, sex, weight, and age (**Supplementary Figure 1A**). Animals were primed with MVA encoding HIV-1 *gag-pol* and *env* from multiple subtypes at month 0 and boosted at months 3, 6, and 12 with MVA plus adjuvanted gp145 (CO6980v0c22, subtype C) adjuvanted with either ALFA or aluminum hydroxide (alum). Control animals received MVA lacking HIV-1 inserts and ALFA adjuvant alone. At month 15 macaques were serially challenged intrarectally every other week with SHIV-1157ipd3N4 (AID40) until viremic for up to ten challenges. Immune responses to vaccination were assessed in all three arms at five pre-challenge time points, including months 0, 3, 3.5, 6.5, and 12.5, and at first and sixth challenges (**Supplementary Figure 1B**).

### Fc Array

Fc and Fv characteristics of antigen-specific sera polyclonal antibodies raised in response to the vaccines and challenges were evaluated using an Fc array assay (8). Briefly, a panel of thirty-seven recombinant SHIV/HIV-1 proteins were covalently coupled to fluorescent beads. Sera were analyzed at a dilution of 1:1,000 for detection reagents specific for tetramerized rhesus Fcγ receptor (FcγR2A-2, FcγR2A-3, FcγR2A-4, FcγR2B-1, FcγR3A-1, and FcγR3A-3) and human Fcγ receptor (FcγR2aH, FcγR2aR, FcγR2b, FcγR3aF, FcγR3aV, and FcγR3b NA1) detection reagents, whereas the dilutions used for analysis with rhesus IgG (Southern Biotech #6200-09, polyclonal, Lot B0112-YC26B) were 1:1,000 and 1:500. For aHu IgA (Southern Biotech #2050-09, polyclonal, Lot C5213-XA55X) and C1q, the dilution used was 1:250. The optimal serum dilution factors were determined experimentally (9). Beads were first incubated with antibodies, washed, and incubated with Fc detection reagents. Plates were subsequently washed and Median Fluorescence Intensity (MFI) data were collected using an array reader. Prior to analysis, Fc array data were filtered for quality control using a three-step process. First, coefficients of variation (CV) were calculated for all intra-plate sample replicates. The replicates leading to poor reproducibility (CV > 0.15) were identified and excluded. Second, MFIs below 45 were marked as out of range and excluded. In cases where both replicates had MFIs below 45, these low values were presumed to be correct, and a value of 40 was assigned. Third, the Z-factor was applied to determine whether an Fc array measure has a positive signal, which is a measure of a signal quality using the concept of a separation band between background (pre-immune) and sample (post-immune) signals (10). Fc array data with non-positive signals were excluded.

### Data Analysis

**Figure 1:**
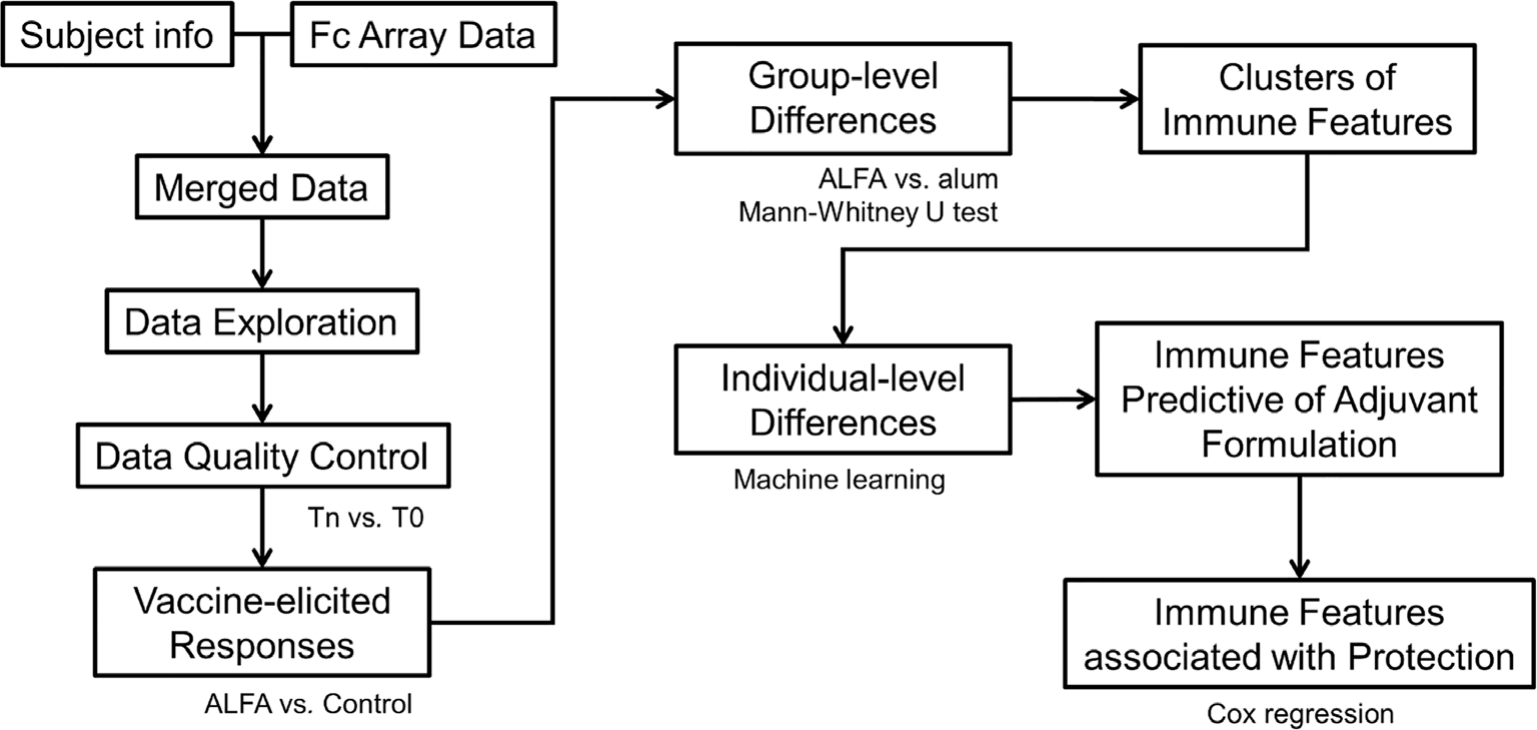
Data analysis pipeline.

Vaccine-elicited immune responses (features) were determined using univariate analysis. Each immune response was compared to its pre-immune reference point and its reference in the Control arm, respectively. Identified vaccine-elicited immune responses were then compared between two vaccination arms, ALFA and alum, to identify differences at the group (adjuvant) level. Following univariate analysis, machine learning (e.g., random forest) was performed to determine how well subjects from the two vaccination arms could be distinguished at the individual level and which combination of immune responses contributed most to the distinction. Finally, Cox regression was used to determine if the immune responses most predictive of adjuvant were also correlated with reduced risk to infection that was observed in the ALFA arm (**Figure 1**).

*Univariate analysis*. To identify vaccine-elicited immune responses, univariate analysis for each immune measure was performed by comparing post-immune with pre-immune responses. Then, each post-immune response was compared with the corresponding measure from the Control arm. Wilcoxon signed-rank test and Mann-Whitney U test were used to calculate statistical significance, respectively (11). Immune measures in which comparisons to the pre-immune and Control data both showed a significant difference at p-value < 0.05 were selected as vaccine-elicited immune responses. Vaccine-elicited immune responses were further compared between the two adjuvant arms (ALFA vs. alum) using the Mann-Whitney U test to determine group-level difference with respect to adjuvant. To control the false discovery rate (FDR), resampling-based FDR adjustments were employed (12). One thousand permuted datasets were created by randomly shuffling the label (adjuvant) of each subject in the two vaccination arms. For each permuted dataset, the smallest p-value of the Mann-Whitney U tests across all comparisons was selected to create a probability distribution for the 1,000 lowest p-values obtained by random chance. The corrected p-value was calculated by comparing where the uncorrected p-value lies in the permuted distribution of the 1,000 lowest p-values. Adjuvant-associated differences were determined by identifying the vaccine-elicited immune responses that showed a significant difference between ALFA and alum arms at a p-value < 0.05 and a q-value < 0.2.

*Multivariate analysis and machine learning*. Spearman correlation coefficients between immune measures were calculated to create correlation matrices (13). Correlated immune measures were further clustered using hierarchical clustering (14). The optimal number of clusters was determined using the elbow method. Medoids in each cluster were identified as representative immune measures. The random forest approach was applied to build machine learning models to predict adjuvant arms using immune measures (15). Model training and parameter tunings were carried out using repeated 5-fold cross-validation, subsampling the data set by 5-fold and resampling 100 times. The hyperparameter, mtry (number of variables randomly sampled as candidates at each split), was adjusted to identify the optimal out-of-bag error, an unbiased estimate of the generalization error. To evaluate the predictive accuracy of the RF modeling approach, cross validation were utilized, where data samples were subsampled by bootstrap aggregating for training and prediction performance was evaluated on those observations that were not used in training. Model performance was expressed as both a percentage of correctly predicted outcomes with a Cohen’s kappa value (16), and as the area under the curve of the receiver operating characteristic (AUCROC) (17). Cohen’s kappa statistic is an unbiased measure for imbalanced class problems. To assess the statistical significance of the RF models and ascertain overfitting that might occur in the machine learning process, AUCROC-based permutation tests were carried out (18). In permutation tests, the labels (i.e., adjuvant type) of the training data were shuffled randomly. Random forest models were then rebuilt using the data with permuted labels 100 times. AUCROC was computed to evaluate prediction performance of permutation models. Based on the AUCROC of the permutation models, null distributions for AUCROC were also estimated.

*Survival analysis*. Survival analysis was used to investigate the time it takes for a subject to get infected by SHIV (19). The discrete infecting challenge was considered as the time to infection. Subjects that had not been infected by the tenth challenge were treated to be censored. Kaplan–Meier plots were created to visualize survival/time-to-event curves and log-rank test was used to compare the survival/time-to-event curves of two arms. Cox proportional-hazards model was fit to investigate the effect of immune measures on time to infection (20).

All statistical analyses were performed using the R stats package and machine learning were carried out using the R caret package.

## Results

### Pox-Protein Vaccine Efficacy in Rhesus Macaques

To improve HIV-1 vaccine immunogenicity and efficacy, we recently evaluated pox-protein vaccination using a next generation liposome-based adjuvant, ALFA, in rhesus monkeys (6). It was found that SHIV infection risk trended lower with ALFA-adjuvanted vaccination relative to controls, while no vaccine efficacy was observed in the alum arm (**Supplementary Figures 1A–D**).

### Fc Receptor-Related Immune Responses Elicited by ALFA-Adjuvanted Vaccine

**Figure 2:**
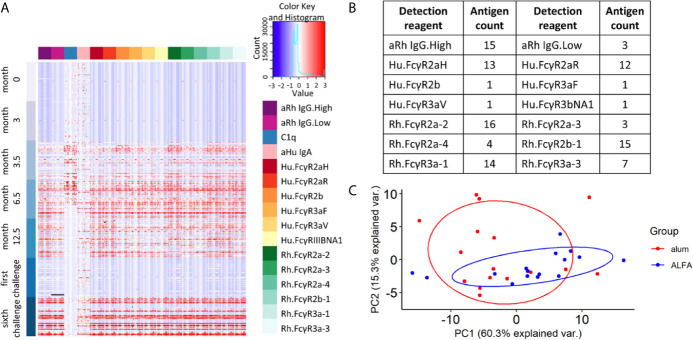
Vaccine-elicited and Fc-mediated effector function. **(A)** Heat map for all Fc array measurements performed on all 48 study animals (rows) at each study time point by HIV-1 Env antigen and immune features (columns). **(B)** Fc features of vaccine-elicited immune responses at 12.5 months post-vaccination. **(C)** Principal component analysis on vaccine-elicited immune responses at 12.5 months post-vaccination by active vaccine group.

To investigate the underlying mechanism(s) linked to the observed efficacy of ALFA-adjuvanted vaccination against SHIV acquisition, Fc array data analyses were performed to characterize the vaccine-elicited, HIV-1 Env-specific antibody effector profiles. Exploration of the whole data set showed that most effector immune responses appeared after the first boost (month 3.5), were sustained or increased with subsequent boosts, and decayed by the time of the first SHIV challenge (month 15). C1q-mediated immune responses declined faster by month 12.5, while IgA responses were limited relative to pre-immunization baseline values (**Figure 2A**). Vaccine-elicited immune responses were determined by comparing each immune response to its pre-immune and control arm values as reference. The broadest range of Fc receptor-related immune responses was identified at month 12.5, two weeks post the last immunization. We identfieid 106 vaccine-elicited immune respones in the active arms at this peak immunogenicity time point and these respones were captured by 14 detection reagents (**Figure 2B**). Comparison of these responses between ALFA and alum arms by PCA was unable to discriminate animals by adjuvant group (**Figure 2C**), indicating that vaccine-elicited immune responses with large variance may not be associated with adjuvants.

### Differential Fc Receptor-Related Immune Responses by Adjuvant

**Figure 3:**
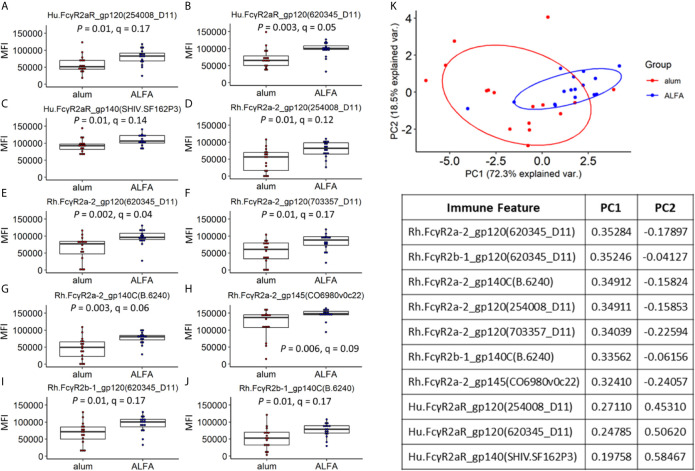
Vaccine-elicited immune responses that vary by adjuvant. **(A–J)** Vaccine-elicited binding antibody responses differing between the ALFA and alum active arms are shown for each Fc detection reagent and HIV-1 Env antigen combination. MFI, median fluorescence intensity. **(K)** Principal component analysis on adjuvant-associated immune responses.

Because the main aspects of variation in the data did not relate to adjuvants, yet differential protection was observed between adjuvants, we next sought to identify differences in individual immune features that were associated with adjuvants. Univariate analyses of the Fc receptor-related immune responses were performed by comparing each vaccine-elicited immune response between the ALFA and alum active arms. While all twelve Fcγ receptors characterized in the Fc array were represented among the vaccine-elicited responses, the two adjuvants arms differed only in ten responses related to just three Fcγ receptors: Hu.FcγR2aR, Rh.FcγR2a-2, and Rh.FcγR2b-1 (**Figures 3A–J**). Fcγ receptors 2a and 2b are close homologs known to be responsible for phagocytosis (21, 22). These ten vaccine-elicited immune responses were also compared between the ALFA and alum active arms using PCA, which distinguished animals by adjuvant when using this subset of vaccine-elicited immune responses (**Figure 3K**). The two principal components, PC1 and PC2, captured over 90% of the variation in the data set. By comparing the PCA plot with the one in **Figure 2C**, we found that the overlap between ALFA and alum clusters is smaller in **Figure 3K**, which indicates that the 10 adjuvant-associated immune responses have stronger classification power for separating adjuvant groups.

### Fcγ Receptor 2a-Related Immune Responses Most Predictive of Vaccine Adjuvant

**Figure 4:**
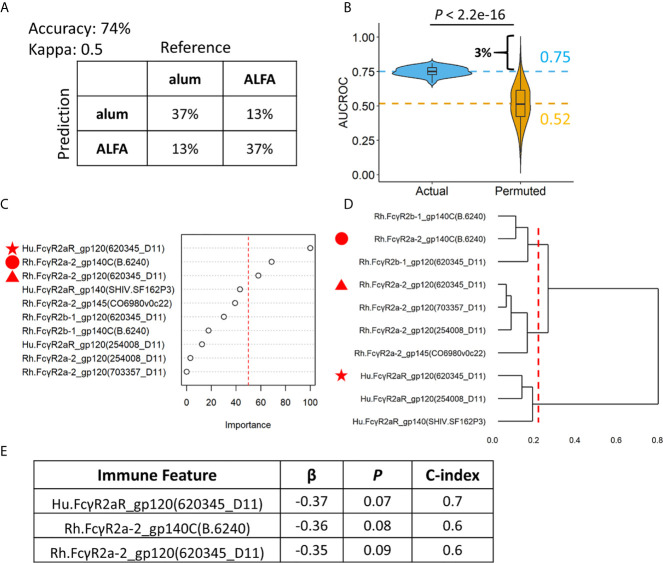
Random forest models revealing individual level differences between ALFA and alum. **(A)** Prediction accuracy, kappa, and confusion matrices. The rows of confusion matrices represent the predicted adjuvant arms, whereas the columns indicate the actual adjuvant arms. **(B)** Comparison of AUCROC values from 100 repetitions of 100 times repeated 5-fold cross-validation using actual (blue) versus permutated (yellow) adjuvant labels. Dashed line represented the mean AUCROC values. **(C)** Immune feature importance in random forest models. **(D)** Hierarchical clustering on immune features used in random forest models. **(E)** Cox regression analysis on three most important immune features identified by random forest models.

Machine learning was applied to make an individualized assessment of adjuvant-associated effects. A set of random forest models were built (n=100) to predict whether each animal in the active arms received ALFA- or alum-adjuvanted vaccine based on the ten identified vaccine-elicited immune responses that differed between adjuvant groups. We assessed model prediction performance using repeated cross validation and permutation tests. The confusion matrix created from the results of 100 repeated 5-fold cross-validations of the data showed that the random forest model achieved an accuracy of 74%, and a kappa value of 0.50, indicating moderate to strong predictive performance (16) (**Figure 4A**). In order to assess the overfitting, the random forest model was applied using a permutation test whereby the labels (adjuvants) of the data were randomly shuffled. The AUCROC of models built with the randomly shuffled data was 0.52, which was significantly lower than the average AUCROC of actual models, 0.75 (**Figure 4B**). The permutation test also revealed that there was only a 3% probability that the AUCROC of actual models, 0.75, could be obtained at random. The average AUCROC of models built with the randomly shuffled data was close to 0.5, indicating that our model was not overfitted. The importance of the ten vaccine-elicited immune responses that were employed in the random forest model was measured using relative importance scores (**Figure 4C**). We found that three Fcγ receptor 2a-related immune responses were most predictive of vaccine adjuvant: Hu.FcγR2aR_gp120(620345_D11), Rh.FcγR2a-2_gp140C(B.6240), and Rh.FcγR2a-2_gp120(620345_D11). These three immune responses belong to three different clusters defined by hierarchical clustering of the ten immune responses employed in the random forest model (**Figure 4D**). The association between these three immune responses and infection risk was investigated using the Cox proportional-hazards model and the pooled data from ALFA and control arms. All three of these responses were negatively associated with infection risk at a significance level of 0.1 (**Figure 4E**). This finding supports an FcγR2a-mediated Env-specific binding antibody-dependent mechanism underlying the protection observed with ALFA-adjuvanted vaccination. Fcγ receptor 2a is known to be responsible for executing phagocytosis, which independently correlated with protection (6).

### Sex-Differential Effect of ALFA-Adjuvanted Vaccine

**Figure 5:**
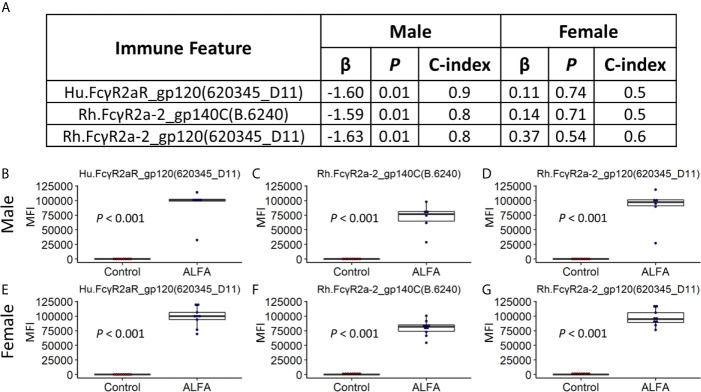
Sex-specific efficacy and vaccine-elicited immune responses. **(A)** Cox regression analysis for Male and Female subjects on three most important immune features identified by random forest models. **(B–D)** Vaccine-elicited immune responses of Male subjects in the ALFA arm. **(E–G)** Vaccine-elicited immune responses of Female subjects in the ALFA arm. MFI, Median Fluorescence Intensity.

Sex-based differences in vaccine responses are well established both in humans and animal models (23, 24). Two striking sex differences were observed in this macaque study: 1) ALFA-mediated vaccine efficacy was limited to male animals; and 2) the infection rate in females was much lower than that of males, independent of vaccine group (6) (**Supplementary Figures 1E–F**). We explored immune responses associated with challenge outcomes stratified by sex. Using Cox proportional-hazards modeling, the three Fcγ receptor 2a-related immune response features most predictive of vaccine adjuvant at month 12.5 were also negatively associated with infection risk in males at a significance level of 0.01 (**Figure 5A**). However, sex differences in the magnitude of these three immune responses most predictive of adjuvant were not identified, as both males and females immunized with ALFA-adjuvanted vaccine mounted similarly robust responses (**Figures 5B–G**). Therefore inherent sex-based differences in vaccine immunogenicity did not appear to contribute to the discordant challenge outcomes between vaccinated males and females.

## Discussion

RV144 and several recent NHP vaccine studies have shown evidence that antibody effector activities are associated with reduced risk to HIV/SHIV infection, highlighting a protective role of non-neutralizing antibodies for HIV vaccine design (22, 25–28). Thus, there is growing interest in studying non-neutralizing Fc functional antibodies and their contributions to novel correlates of protection. The Fc array was applied in this study to capture multi-dimensional profiles of Fc effector functions. We compared antigen-specific binding antibody Fc array immunoprofiles of rhesus macaques enrolled in a pox-protein HIV-1 vaccine efficacy study in which protein was adjuvanted with either conventional alum or ALFA. Characterizing immunoprofiles of adjuvanted vaccines and identifying their immune signatures may aid in understanding protective mechanisms modulated by adjuvants and identifying appropriate vaccine adjuvant(s) for specific pathogens. We found that adjuvanting with ALFA induced stronger Fcγ receptor 2a-related binding antibody responses and these responses were associated with protection against SHIV acquisition.

Fcγ receptor 2a is a cell surface receptor that is expressed on phagocytic cells, such as macrophages, monocytes, neutrophils, and dendritic cells, and involved in phagocytosis. Genetic variations of Fcγ receptor 2a correlate with progression of HIV infection (29), susceptibility to perinatal HIV-1 infection (30), and HIV vaccine effects (31). In addition, Fcγ receptor 2a and 2b-related immune responses have been found to correlate with the phagocytic activity of HIV-specific antibodies (21, 22). Our study not only confirms the findings of the previous study, i.e., that the ALFA-adjuvanted vaccine enhanced induction of phagocytic responses as assessed by functional assays (6), but we can also infer a potential phagocytic mechanism of the ALFA-adjuvanted vaccine, which involves the Fcγ receptor 2a.

Previous studies have shown that females often mount greater antibody responses to immunization or infection than males (32–34), but sex differences in terms of non-neutralizing effector antibody responses have not been investigated in depth. In the present study, we evaluated the sex-differential effect of ALFA-adjuvanted vaccine. The ALFA-adjuvanted vaccine elicited Fcγ receptor 2a-mediated humoral immune responses that were positively correlated with protection, but only in males. Future, well-powered studies including both sexes will be valuable to further identify sex-based differences in vaccine outcomes and immune correlates.

Systems serology is a relatively new data-driven approach that can analyze high-throughput experimental data to comprehensively survey a diverse array of antibody features and functions. This information can be used to identify new correlates of protection from infection and lead to a more comprehensive understanding of vaccine mechanisms that underlie protection (35, 36). Systems serology has been applied to search for immune features that best predict protection induced by HIV vaccines (37–39), malaria vaccines (40–42), and other vaccines (43, 44). In the present study, we developed a systems serology pipeline that integrated both machine learning and Cox proportional hazards regression to analyze high-dimensional Fc array data. We found that antibody-dependent Fcγ receptor 2a-related effector functions were augmented by the ALFA adjuvant, and these responses were associated with enhanced protection in male animals. Our results highlight how systems serology can be used to identify biological mechanisms that underlie vaccine-induced protection. Furthermore, the analyses showcase how to use in-depth statistical analysis of complex data to advance the study and exploration of next generation adjuvants aimed at developing a globally effective HIV vaccine.

## Data Availability Statement

The data used in this article can be made available upon request. Requests to access these datasets should be directed to DB, dbolton@hivresearch.org.

## Author Contributions

PL, SC, DB, and AW designed the project. DG, SL, MA, and DB produced the data. PL performed the data analyses. PL and DB wrote the manuscript. DG, SL, SC, MA, DB, and AW reviewed and edited the manuscript. All authors contributed to the article and approved the submitted version.

## Funding

This work was supported by the U.S. Army Medical Research and Development Command under Contract No. W81XWH20C0031, by the Military Infectious Disease Research Program and by a cooperative agreement (W81XWH-11-2-0174) between the Henry M. Jackson Foundation for the Advancement of Military Medicine, Inc. (HJF), and the U.S. Department of Defense (DOD).

## Disclaimer

The opinions and assertions contained herein are the private views of the authors and are not to be construed as official or as reflecting the views of the U.S. Army, the U.S. Department of Defense, or The Henry M. Jackson Foundation for the Advancement of Military Medicine, Inc. (HJF). This manuscript has been approved for public release with unlimited distribution.

## Conflict of Interest

The authors declare that this study received funding from the Henry M. Jackson Foundation for the Advancement of Military Medicine, Inc. The funder was not involved in the study design, collection, analysis, and interpretation of data, the writing of this article or the decision to submit it for publication.
