# Supplementary material for: Immunoprofiling Correlates of Protection Against SHIV Infection in Adjuvanted HIV-1 Pox-Protein Vaccinated Rhesus Macaques
Source: Front Immunol. 2021 May 11;12:625030. doi: 10.3389/fimmu.2021.625030 (PMC8144500; doi:10.3389/fimmu.2021.625030)
Supplement: Supplementary file 1 [file DataSheet_1.pdf]

Supplementary Material

Supplementary Figures

A

Group	N*	Prime	Boost	Adjuvant
alum	16	MVA-A/C/E	gp145-C trimer	Aluminum hydroxide (AH)
ALFA	16	MVA-A/C/E	gp145-C trimer	ALFA (MPLA + AH)
Control	16	empty MVA	None	ALFA (MPLA + AH)

*Indian-origin rhesus macaques balanced for gender, age, weight, and *TRIM* alleles

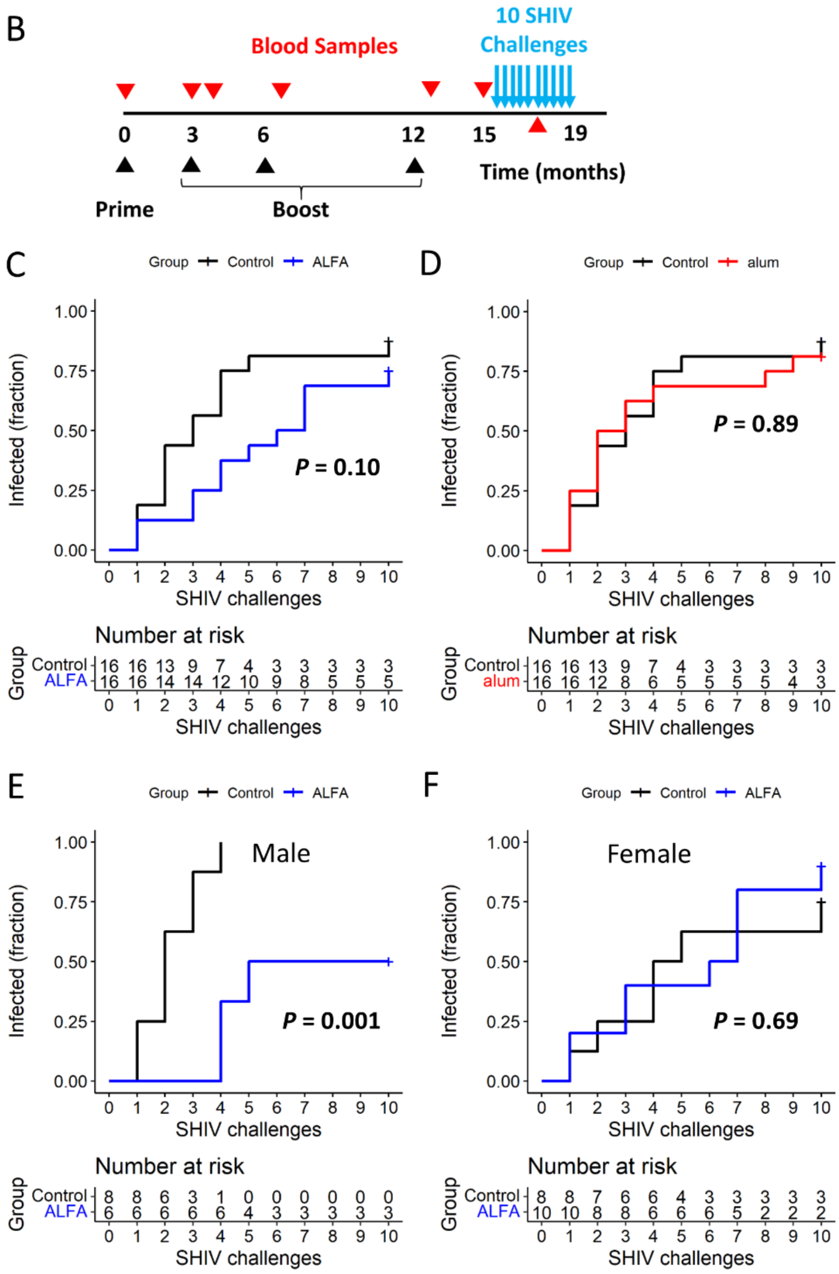

Supplementary Figure 1. Study design and SHIV challenge outcome. (A) Study arms and vaccine regimens. (B) Study design and schedule. (C-D) SHIV infection acquisition curves displayed separately for alum and ALFA arms. (E-F) SHIV infection acquisition curves displayed separately for male and female subjects in the ALFA arm.
